# Virus-induced gene silencing simultaneously exploits ‘attract and kill’ traits in plants and insects to manage huanglongbing

**DOI:** 10.1093/hr/uhae311

**Published:** 2024-11-06

**Authors:** Nabil Killiny, Yasser Nehela, Subhas Hajeri, Siddarame Gowda, Lukasz L Stelinski

**Affiliations:** Department of Plant Pathology, Citrus Research and Education Center, IFAS, University of Florida, 700 Experiment Station Road, Lake Alfred, FL 33850, United States; Department of Agricultural Botany, Faculty of Agriculture, Tanta University, Seberbay, Tanta, Gharbia 31527, Egypt; Citrus Pest Detection Program, Alliance of Pest Control Districts, 22847 Road 140, Tulare, CA 93274, United States; Department of Plant Pathology, Citrus Research and Education Center, IFAS, University of Florida, 700 Experiment Station Road, Lake Alfred, FL 33850, United States; Department of Entomology and Nematology, Citrus Research and Education Center, IFAS, University of Florida, 700 Experiment Station Road, Lake Alfred, FL 33850, United States

## Abstract

The vector-borne disease huanglongbing (HLB) causes severe economic losses to citrus production worldwide with no available cure. Herein, we applied virus-induced gene silencing technology to engineer citrus that preferentially attracted and specifically killed *Diaphorina citri*, the vector associated with HLB. We engineered the infectious citrus tristeza virus (CTV-T36) clone to carry three truncated genes. The triple construct (CTV-t*Awd*-t*Wnt*-t*PDS*) produces small interfering RNAs (siRNAs) against phytoene desaturase, *PDS*, to yield a phenotype with visual, olfactory, and gustatory cues that preferentially attracted *D. citri.* In addition, siRNAs targeted two genes related to flight in *D. citri*, abnormal wing disc (*DcAwd*) and wingless (*DcWnt*), that caused wing malformations and decreased survival in psyllids that fed on plants inoculated with the engineered virus*.* During two successive generations, *D. citri* reared on CTV-t*Awd*-t*Wnt*-t*PDS*-inoculated plants exhibited higher mortality across life stages as well as reduced fecundity and fertility as compared with those reared on noninfected plants or CTV-wt-inoculated plants. Furthermore, CTV-t*Awd*-t*Wnt*-t*PDS*-inoculated plants shortened the lifespan of *D. citri* by more than 20 days. Morphological abnormalities were noted in those adults that did successfully emerge on plants inoculated with CTV-t*Awd*-t*Wnt*-t*PDS*, including cocked wings with a bowl-shaped depression and/or a convex shape. Phloem sap from CTV-t*Awd*-t*Wnt*-t*PDS*-inoculated plants decreased the survival of *D. citri* adults, confirming that siRNAs were present in the sap of these plants. Collectively, we provide proof of concept for a novel variant of the attract-and-kill method where the cultivated crop is potentially transformed into a hyper-attractive population and transmission sink for a phytopathogen vector.

## Introduction

Since the initial detection of huanglongbing (HLB, aka citrus greening disease) in Florida in 2005, it has spread rapidly throughout the state, resulting in widespread tree decline, reduced fruit quality, and significant yield losses leading to severe economic decline in citrus-producing regions [1]. HLB is a vector-borne disease that is epidemiologically determined by the interactions among the susceptible host, insect vector, virulent pathogen, and favorable environmental factors [2, 3]. Although Koch’s postulates have not yet been fulfilled for HLB due to the challenges in culturing the putative bacterial pathogen, the association between HLB and a fastidious, phloem-limited, and phytopathogenic bacterium, *Candidatus *Liberibacter**spp., is well established [4–7].

Taxonomically, three bacterial species have been associated with HLB, including ‘*Ca. *L. asiaticus**’ in the Arabian Peninsula, Africa, Asia, and the Americas; ‘*Ca. *L. americanus**’ in Brazil; and, ‘*Ca. *L. africanus**’ in Africa [2, 6–11]. Among these three species, ‘*Ca. *L. asiaticus**’ is dominant and responsible for causing cumulative economic losses exceeding billions of dollars to the Florida citrus industry in particular, and citrus production worldwide in general has endured mounting annual losses [6, 7]. The tree-to-tree transmission of ‘*Ca. *Liberibacter** spp.’ can occur via graft inoculation; however, the pathogen is mainly transmitted by citrus psyllid species [12]. The African citrus psyllid, *Trioza erytreae* Del Guercio (Hemiptera: Triozidae), transmits ‘*Ca. *L. africanus**’ [6, 13], and the Asian citrus psyllid, *Diaphorina citri* Kuwayama (Hemiptera: Liviidae), transmits both ‘*Ca. *L. asiaticus**’ and ‘*Ca. *L. americanus**’ [6, 14, 15].

Unfortunately, HLB has no sustainable cure yet [6], and disease management programs rely on integrated pest management approaches, which combine cultural practices, biological control, chemical control, and emerging technologies [16–20]. Moreover, current management practices for citrus infected with HLB now also incorporate applications of broad-spectrum antibiotics [20–24]. These inputs are not sustainable in regions where tree infection rates are high because yields from trees with HLB disease do not generate sufficient profit to cover production costs [25]. Extensive use of insecticides and/or antibiotics in citrus orchards contaminates soil and water, exceeding maximum residue levels. Additionally, it may lead to the emergence of insecticide-resistant populations of *D. citri* [26]. Collectively, and due to the increased awareness of environmental protection, the identification of eco-friendly alternative management technologies has become increasingly accepted. Emerging technologies to control HLB include antimicrobial peptides that kill ‘*Ca. *L. asiaticus**’ or induce citrus immunity [27, 28] as well as repellants [19, 20] and attract-and-kill devices [29–32] to reduce populations of the vector.

Like most phytophagous insects, *D. citri* use several sensory modalities to locate and accept their host plants [16]. This can be exploited to develop attract-and-kill devices that broadcast specific visual, olfactory, and in some cases, gustatory cues that mimic natural hosts [33–35]. Attract-and-kill typically combines these attractants with a killing agent on a substrate. The killing agent must consist of a fast-acting contact insecticide to eliminate insects attracted to the target because contact with the target is brief [36–38]. These devices can be individual targets that are hung in trees, or in some cases take the form of netting that must be deployed by hand onto the crop [35, 36]. Conventional attract and kill using artificial bait stations deployed in citrus trees can reduce populations of *D. citri* [38]. These devices combine visual cues (i.e., yellow color) with a contact toxicant (β-cyfluthrin) applied to a landing platform where the insect obtains a sublethal or lethal dose of toxicant during contact [38]. Importantly, attract-and-kill technologies are highly sensitive to pest density, with efficacy decreasing as pest densities increase [39], unlike insecticides, which operate quite independently of pest density. However, *D. citri* is a highly r-selected pest [40] and thus occurs at very high population densities per area of crop [41]. Although incremental improvements have been made to both the visual [42, 43] and semiochemical [29, 44] cues associated with artificial attract-and-kill technologies targeting *D. citri* to increase vector attraction, application of attract and kill for HLB management has failed to date because the number of devices required per area of crop to achieve efficacy is impractically high [43].

Previously, we proposed the possibility of an attract-and-kill strategy by silencing a phytoene desaturase gene in citrus with dsRNA delivered using a *citrus tristeza virus* (CTV)-vector [30]. Briefly, CTV-delivered, phytoene desaturase silencing (CTV-t*PDS*) yields citrus plants that express visual, olfactory, and gustatory cues that attract *D. citri*. This occurs because silencing the *phytoene desaturase* (*PDS*) gene causes a consistent photobleached phenotype, especially on leaf midribs, veins, and veinlets, that yields attractive visual cues to *D. citri* at close range to cause landing. Interestingly, *D. citri* were more attracted to plants with CTV-t*PDS* than control plants [30]. Furthermore, CTV-t*PDS* plants released volatile organic compounds (VOCs) that were more attractive olfactory cues to *D. citri* than those from control plants when insects had to choose from a distance. Finally, the chemical composition of phloem sap in CTV-t*PDS* plants is enriched as compared with control plants, increasing gustatory cues to stimulate feeding behavior in *D. citri* [30]. Collectively, plants expressing these phenotypic traits attract *D. citri* and could yield a trap plant if combined with RNAi-inducing truncated genes (double constructs) that could reduce the survival or fitness of *D. citri*. Previous targets for CTV-based delivery of RNAi to *D. citri* have included cathepsin B and L that disrupted ovarial development when silenced [45]. Furthermore, CTV-based silencing of cytochrome P_450_ genes increases susceptibility of *D. citri* to toxins [46]. Although these examples prove the concept of delivering dsRNA to psyllids via the plant using a CTV vector, neither of these gene targets are compatible with attract and kill.

In contrast, abnormal wing disc (*Awd*) is a strong candidate for gene silencing to achieve attract and kill because it could reduce populations of *D. citri*. Silencing *Awd* in *D. citri* nymphs *in vitro* with topically applied dsRNA significantly increased mortality, disrupted wing development, and reduced emergence of adults [47]. Moreover, *D. citri* nymphs feeding on *Citrus macrophylla* expressing a truncated *D. citri Awd* gene delivered by a CTV-RNAi vector significantly decreased transcript levels of *Awd* in *D. citri* nymphs and caused a malformed-wing phenotype in adults, which impaired flight and decreased survival [48]. Impairing flight in *D. citri* adults would potentially limit tree-to-tree transmission of the ‘*Ca. L. asiaticus*’ bacterium in citrus orchards.

Likewise, the wingless (*Wnt*) pathway is a well-characterized intercellular signaling network that plays multiple functions during both embryogenesis and adult homeostasis in *Drosophila* [*49–54**]*. For example, *Wnt* supplies a key signal for pre- and postsynaptic differentiation [52], cell proliferation and polarity, as well as the specification of cell fate in *Drosophila* [50, 53]. Moreover, *Wnt Drosophila* mutants are characterized by abnormal segment polarity and limb development [49]. Similarly, *Dwnt*-2 *Drosophila* mutants exhibit defects in muscle and testis development indicating that *DWnt-2* is essential for the development of male reproductive organs [51], whereas *Wnt6* is required for maxillary palp formation [54]*.* It is worth mentioning that genes involved in the *Wnt* signaling pathway of *D. citri* were recently characterized and annotated using a chromosomal-length genome assembly [*55**[.* Briefly, the *D. citri* genome possesses 24 *Wnt* signaling genes with high homology for beta-catenin, Frizzled receptors, and seven Wnt ligands (*Wg/Wnt1*, *Wnt5*, *Wnt6*, *Wnt7*, *Wnt10*, *Wnt11*, and *WntA*) [*55*]*.* However, the exact role(s) of these *Wnt* genes in *D. citri* has not yet been resolved.

In the current investigation, we engineered a triple construct of CTV-RNAi to infect citrus plants that includes (1) a truncated *PDS* from citrus to yield visual, olfactory, and gustatory cues in the infected plant to attract *D. citri* as well as (2) *Awd* and *Wnt* genes that yield flight-related and physiological defects in those *D. citri* that feed on the plant*.* We suggest that citrus trees expressing this triple construct (CTV-t*Awd*-t*Wnt*-t*PDS*) within *D. citri*-infested orchards could function to specifically attract and eliminate the pathogen vector. This innovative approach could be implemented in an economically viable manner within citrus industries affected by HLB. It aligns well with ecologically sustainable methods, including conservation biological control, enabling growers to sustain the health and productivity of their orchards amidst prevalent HLB by decreasing psyllid populations within affected regions. Moreover, this approach aims to decrease reliance on chemical insecticides, thus reducing environmental burdens and associated costs within HLB-infected citrus groves.

## Results

### CTV-t*Awd*-t*Wnt*-t*PDS*-induced gene silencing in citrus

**Figure 1:**
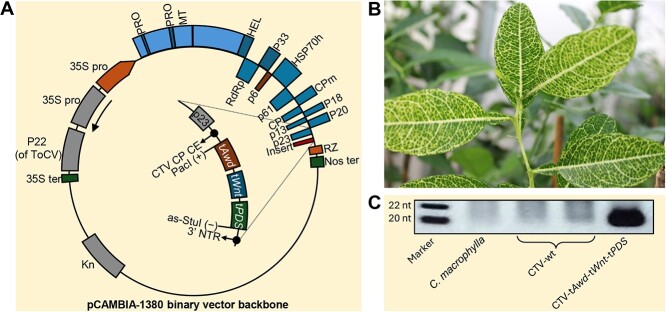
*Citrus tristeza virus* (CTV)-induced gene silencing targeting three genes: an endogenous gene from citrus (*PDS*) and two exogenous genes targeting *D. citri* (*DcWNT* and *DCAWD*). **(A)** Schematic representation of full-length infectious cDNA clones of CTV-t*Awd*-t*Wnt*-t*PDS* in the binary vector pCAMBIA-1380. **(B)** The visible phenotype in citrus plants inoculated with CTV-t*Awd*-t*Wnt*-t*PDS PDS* (typical photobleaching on leaves and stems). **(C)** Northern blot shows accumulation of *PDS, Awd, and Wnt*-specific siRNAs in *C. macrophylla* inoculated with CTV-t*Awd*-t*Wnt*-t*PDS* in comparison to no accumulation observed in *C. macrophylla* CTV-wt plants or noninoculated *C. macrophylla* (controls). SiRNA size markers (M) were designed using synthetic 5′-DIG-labeled oligonucleotides of 18 and 21 mer, which ran as 20 and 22 nucleotides, respectively. Hybridization of the blot was carried out using a digoxigenin-labeled antisense riboprobe that targeted sequences of the *PDS, Awd,* and *Wnt* genes.

Citrus plants were inoculated with a binary plasmid CTV vector consisting of 968 nucleotides with truncated fragments of two genes from *D. citri* (t*Awd* [263 nucleotides] and t*Wnt* [313 nucleotides]), as well as an endogenous gene from citrus (t*PDS* [392 nucleotides]) inserted using unique *Pac*I and *Stu*I restriction sites (Fig. 1A). Wild-type CTV (CTV-wt) was used as a control. *C. macrophylla* plants inoculated with the CTV-t*Awd*-t*Wnt*-t*PDS* construct exhibited a photo-bleached phenotype in newly emerging leaves (Fig. 1B). Northern blot analysis confirmed the accumulation of small interfering RNAs (siRNAs) specific to t*Awd*, t*Wnt*, and t*PDS* in CTV-t*Awd*-t*Wnt*-t*PDS* triple construct plants, which were absent in CTV-wt control plants (Fig. 1C).

### CTV-t*Awd*-t*Wnt*-t*PDS*-inoculated plants are more attractive to *D. citri* than CTV-wt plants

**Figure 2:**
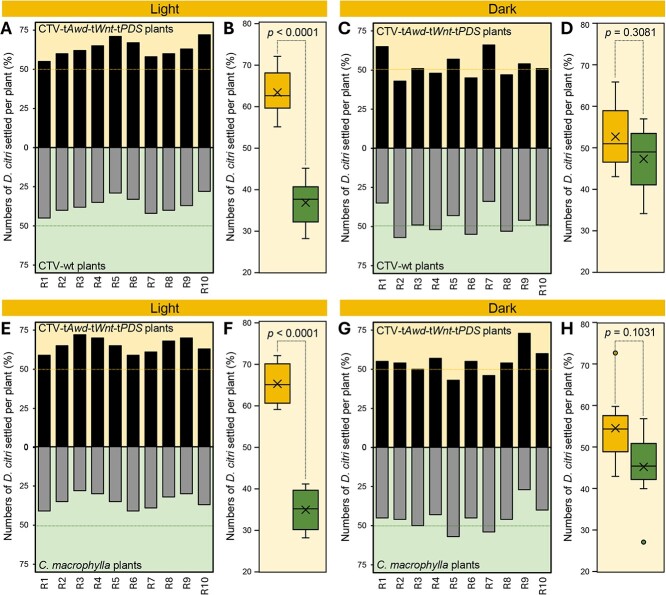
CTV-t*Awd*-t*Wnt*-t*PDS*-inoculated plants attract more *D. citri* than CTV-wt plants. **(A–D)** Settling preference of *D. citri* adults on CTV-t*Awd*-t*Wnt*-t*PDS* versus CTV-wt (control) plants in the presence (light conditions) or absence (dark conditions) of visual cue, respectively. **(E–H)** Settling preference of *D. citri* adults on CTV-t*Awd*-t*Wnt*-t*PDS* versus control *C. macrophylla* in the presence (light conditions) or absence (dark conditions) of visual cue, respectively. In panels A, C, E, and G, whiskers indicate the highest and lowest values of response, horizontal lines show the medians, dots signify the raw data, and boxes show the interquartile ranges. *P* values less than 0.05 indicate statistically significant differences between treatments using the two-tailed *t* test (*P* < 0.05).

Previously, we observed that more *D. citri* adults were attracted to CTV-t*PDS* plants than wild-type controls. The objective of this experiment was to confirm the preference of *D. citri* for CTV-t*Awd*-t*Wnt*-t*PDS*-inoculated versus control plants despite the additional presence of *Awd* and *Wnt* gene constructs. CTV-t*Awd*-t*Wnt*-t*PDS*-inoculated plants attracted more *D. citri* than CTV-wt control plants in 10 separate replicates under light conditions (~63.3 ± 5.5% and 36.7 ± 5.5%, respectively; *P* < 0.0001) (Fig. 2A and B). However, there was no significant difference (*p* = 0.3081) in host selection by *D. citri* between treatment (52.7 ± 7.9%) and control (47.3 ± 7.9%) plants under dark conditions (Fig. 2C and D). Likewise, CTV-t*Awd*-t*Wnt*-t*PDS*-inoculated plants attracted more psyllids than noninoculated *C. macrophylla* under light conditions (65.2 ± 4.7% and 34.8 ± 4.7%, respectively; *P* < 0.0001) (Fig. 2E and F) but not under dark conditions (54.7 ± 8.2% and 45.3 ± 8.2%, respectively; *P* = 0.1031) (Fig. 2G and H). Collectively, these results suggest that visual cues (photobleaching phenotype) associated with CTV-t*Awd*-t*Wnt*-t*PDS*-inoculated plants mediate attraction of *D. citri*.

### CTV-t*Awd*-t*Wnt*-t*PDS*-inoculated plants negatively affect the development of *D. citri*

**Figure 3:**
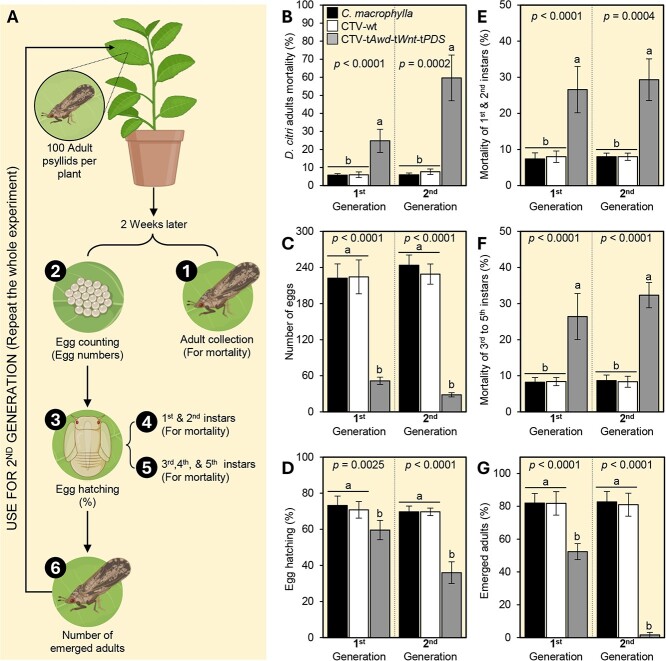
CTV-t*Awd*-t*Wnt*-t*PDS*-inoculated plants negatively affect the development of *D. citri*. **(A)** Schematic representation of the experimental design; **(B)** percentage adult mortality, **(C)** number of eggs laid, **(D)** percentage egg hatch (%), **(E)** percentage mortality of first and second instars, **(F)** percentage mortality of third to fifth instars, and **(G)** percentage adult emergence after rearing psyllids on CTV-t*Awd*-t*Wnt*-t*PDS*-inoculated or control citrus plants for two successive generations. Bars and error bars indicate means and SDs, respectively. Different letters indicate statistically significant differences among treatments, while ‘ns’ indicates no significant differences among treatments using the Tukey–Kramer HSD test (*P* < 0.05).

We evaluated the effect(s) of CTV-t*Awd*-t*Wnt*-t*PDS-*inoculated plants on *D. citri* survival and development by releasing replicate cohorts of newly emerged psyllids on plant treatments and then rearing those over two generations (Fig. 3A). Generally, CTV-t*Awd*-t*Wnt*-t*PDS*-inoculated plants significantly increased mortality of *D. citri* adults and nymphs (Fig. 3B and E), decreased fecundity (Fig. 3C) and fertility (Fig. 3D), and reduced population growth (Fig. 3G).

#### Adult mortality

After 2 weeks of feeding, the percentage mortality of *D. citri* adults on CTV-t*Awd*-t*Wnt*-t*PDS*-expressing plants was significantly greater than that observed on noninfected or CTV-wt-expressing *C. macrophylla* plants during both the first and second generations of exposed insects (Fig. 3B).

#### Fecundity

The number of eggs laid by female *D. citri* on CTV-t*Awd*-t*Wnt*-t*PDS*-expressing plants was significantly lower than that observed on noninfected or CTV-wt-expressing *C. macrophylla* plants during both the first and second generations of exposed insects (Fig. 3C). However, there were no differences between the numbers of eggs laid on the two control treatments (noninfected vs. CTV-wt-expressing *C. macrophylla* plants) (Fig. 3C).

#### Egg fertility

The percentage of viable eggs hatching on CTV-t*Awd*-t*Wnt*-t*PDS*-expressing plants was significantly reduced compared with that observed on noninfected or CTV-wt-expressing *C. macrophylla* plants during both the first and second generations; however, egg viability did not differ between the two control treatments (Fig. 3D).

#### Nymph mortality

Natural mortality of *D. citri* nymphs on the two control treatments (noninfected or CTV-wt-expressing *C. macrophylla*) was below 10% for all nymphal instars during two successive generations (Fig. 3E and F). However, mortality of the first and second instar nymphs (pooled across instars) was significantly greater when psyllids were reared on CTV-t*Awd*-t*Wnt*-t*PDS*-expressing plants than on either control during both the first and second generations of rearing (Fig. 3E). This outcome was consistent with that observed for the third to fifth instars (pooled across instars), with significantly higher mortality observed on CTV-t*Awd*-t*Wnt*-t*PDS*-expressing plants than on either control (Fig. 3F).

#### Adult emergence

The emergence of fully viable *D. citri* adults was never below 80% when nymphs were reared on noninoculated or CTV-wt-expressing *C. macrophylla* control plants (Fig. 3G). However, the percentage of emerging *D. citri* adults emerging on CTV-t*Awd*-t*Wnt*-t*PDS*-expressing plants was reduced to 52.4 ± 4.9% during the first generation of rearing and then dramatically dropped to 1.7 ± 1.5% during the second generation of rearing (Fig. 3G).

### CTV-t*Awd*-t*Wnt*-t*PDS-*inoculated plants reduced the longevity of *D. citri* adults

**Figure 4:**
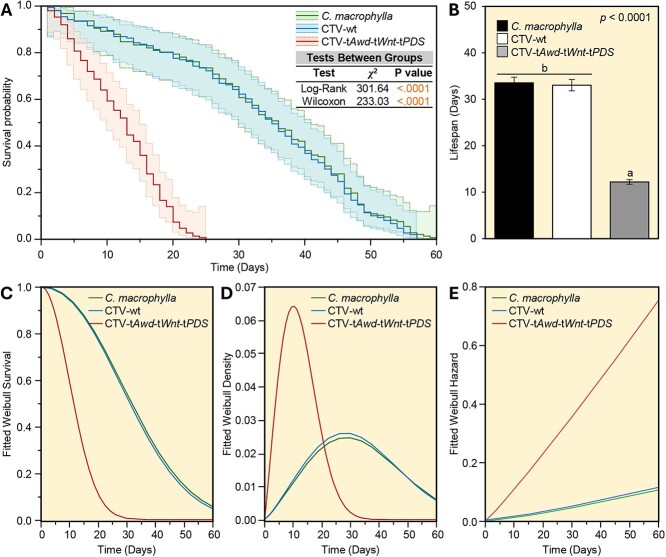
CTV-t*Awd*-t*Wnt*-t*PDS-*inoculated plants reduced the longevity of *D. citri* adults. **(A)** Kaplan–Meier analysis of cumulative *D. citri* survival on noninoculated, CTV-wt-, or CTV-t*Awd*-t*Wnt*-t*PDS*-expressing *C. macrophylla* plants over 60 days (*n* = 50). *P* values and *χ*^2^ of log-rank and Wilcoxon tests (presented within the graph) were used for statistical comparisons among the survival curves. **(B)** Lifespans associated with cumulative survival of *D. citri* on noninoculated, CTV-wt-, or CTV-t*Awd*-t*Wnt*-t*PDS*-inoculated *C. macrophylla* plants over 60 days (*n* = 50). Bars and error bars indicate means and SDs, respectively. Different letters indicate statistically significant differences among treatments, while ‘ns’ indicates no significant differences among treatments using the Tukey–Kramer HSD test (*P* < 0.05). **(C, D, and E)** Fitted Weibull survival, density, and hazard curves, respectively, using the least squares method.

Kaplan–Meier analysis of cumulative survival over 60 days indicated no difference between the survival of *D. citri* adults reared on noninoculated versus CTV-wt-expressing *C. macrophylla* plants (Fig. 4A). However, the probability of *D. citri* survival on CTV-t*Awd*-t*Wnt*-t*PDS*-expressing plants was significantly reduced compared with both controls (*χ*^2^ = 301.64 and *χ*^2^ = 233.03, for log-rank and Wilcoxon tests, respectively, *P* < 0.0001 for both tests) (Fig. 4A). Furthermore, CTV-t*Awd*-t*Wnt*-t*PDS* plants significantly shortened the lifespan of *D. citri* by more than 20 days compared with that observed on controls (Fig. 4B).

To better understand the survival-associated continuous probability distribution, the Weibull regression model was used to evaluate differences in psyllid survival (Fig. 4C–E). The fitted Weibull survival distribution confirmed the above-mentioned findings (Fig. 4C). Briefly, rearing *D. citri* on CTV-t*Awd*-t*Wnt*-t*PDS*-inoculated plants was correlated with a forecasted decline of populations according to the Weibull model. Regression curves can be clustered in two groups: (I) curves that describe the failure rate of *D. citri* under normal conditions (reared on *C. macrophylla* or CTV-wt), which were almost identical, and (II) a curve that describes the failure rate of *D. citri* reared on CTV-t*Awd*-t*Wnt*-t*PDS*-inoculated plants, which were more or less similar to an exponential decay model (Fig. 4C). Moreover, fitted Weibull density curves showed that while *D. citri* reared on noninoculated or CTV-wt-inoculated *C. macrophylla* plants reached their maximum mortality after ~30 days of rearing, psyllids reared on CTV-t*Awd*-t*Wnt*-t*PDS*-inoculated plants reached their peak mortality after only 10 days (Fig. 4D). These findings confirm the predicted Kaplan–Meier-associated lifespan differences between treatments.

Additionally, the Weibull model-associated hazard was analyzed for each treatment and presented in Fig. 4E. It is noticeable that a dramatic change in mortality of *D. citri* adults occurred when populations were maintained on CTV-t*Awd*-t*Wnt*-t*PDS*-inoculated plants as compared with the controls. The failure rate (i.e., hazard rate or force of mortality) accelerated exponentially in *D. citri* populations that were reared on CTV-t*Awd*-t*Wnt*-t*PDS*-inoculated plants, which contrasted with much lower hazard rates observed for psyllids reared on noninoculated or CTV-wt-inoculated *C. macrophylla* plants (Fig. 4E and Table S1).

### CTV-t*Awd*-t*Wnt*-t*PDS*-inoculated plants negatively affect the wing development of *D. Citri*

**Figure 5:**
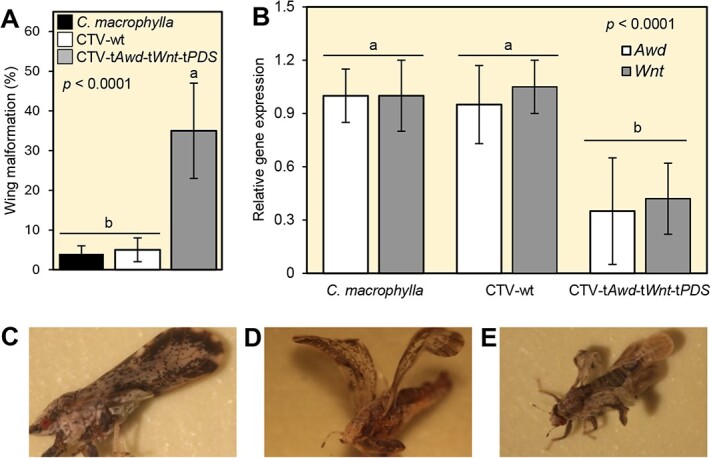
CTV-t*Awd*-t*Wnt*-t*PDS*-inoculated plants negatively affect the wing development of *D. citri*. **(A)** Percentage of *D. citri* emerging with wing malformation and **(B)** relative expression of *DcAWD* and *DcWnt* genes among *D. citri* adults reared on noninoculated, CTV-wt, or CTV-t*Awd*-t*Wnt*-t*PDS*-expressing *C. macrophylla* plants. Bars and error bars indicate means and SDs, respectively. Different letters indicate statistically significant differences among treatments, while ‘ns’ indicates no significant differences among treatments using the Tukey–Kramer HSD test (*P* < 0.05). **(C)** Images of *D. citri* adults emerging from nymphs reared on CTV-wt-inoculated plants, and **(D and E)** different types of wing malformation observed on *D. citri* adults emerging from nymphs reared on CTV-t*Awd*-t*Wnt*-t*PDS*-expressing plants.

Rearing *D. citri* populations on CTV-t*Awd*-t*Wnt*-t*PDS-*inoculated plants resulted in a higher frequency of abnormalities (35 ± 12%) related to wing formation among emerging adults than observed in psyllids reared on noninoculated or CTV-wt*-*inoculated *C. macrophylla* plants where no such malformations were observed (Fig. 5A). Wing malformations were associated with downregulation of both *DcAwd* and *DcWnt* (Fig. 5B), indicating that transcript levels of both genes were significantly decreased in *D. citri* adults reared on CTV-t*Awd*-t*Wnt*-t*PDS*-inoculated plants compared with psyllids reared on control plants (*C. macrophylla* or CTV-wt) (Fig. 5B). Collectively, these findings confirm the acquisition of CTV-specific dsRNAs by *D. citri* feeding on CTV-t*Awd*-t*Wnt*-t*PDS-*inoculated plants*.* The malformations observed in a proportion of psyllids reared on CTV-t*Awd*-t*Wnt*-t*PDS*-inoculated plants occurred in forewings, hindwings, or both (Fig. 5C–E). Silencing of *DcAwd* and *DcWnt* caused numerous types of phenotypic changes to wings, including, but not limited to, cocked wings with a bowl-shaped depression and/or a convex morphology, and in certain cases, prevented successful adult emergence from the fifth instar exuvia (Fig. 5D). In the latter cases, the eclosion of wing-malformed adults was incomplete because the exuvial remained tightly attached to the teneral adult. Some abnormalities were also observed with the dorsal thoracic sclerites (Fig. 5D). Moreover, malformed adults were not able to move normally or fly.

### Treatment of *D. citri* fifth instar nymphs with CTV-t*Awd*-t*Wnt*-t*PDS* phloem sap increased adult mortality

**Figure 6:**
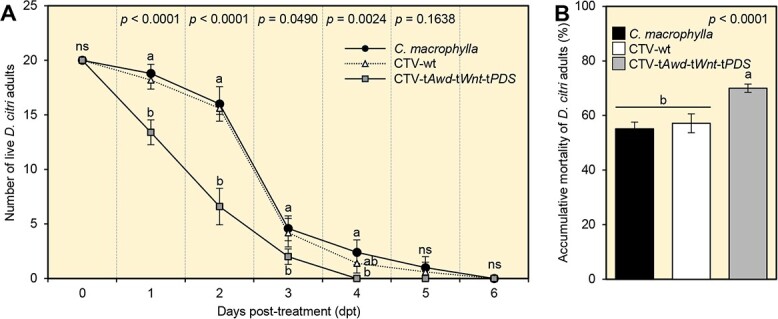
Topical application of CTV-t*Awd*-t*Wnt*-t*PDS* phloem sap increased mortality of *D. citri* adults. **(A)** Number of living *D. citri* adults and **(B)** percentage cumulative mortality of *D. citri* adults reared on noninoculated, CTV-wt-, or CTV-t*Awd*-t*Wnt*-t*PDS*-expressing *C. macrophylla* plants. Values and error bars indicate means and SDs, respectively. Different letters indicate statistically significant differences among treatments, while ‘ns’ indicates no significant differences among treatments using the Tukey–Kramer HSD test (*P* < 0.05).

To confirm the effect of CTV-specific dsRNAs in the phloem sap of CTV-t*Awd*-t*Wnt*-t*PDS*-inoculated plants against *D. citri*, phloem sap was collected from the various plant treatments compared here and then fed to psyllids via topical application. Topical application of phloem sap collected from CTV-t*Awd*-t*Wnt*-t*PDS*-inoculated plants significantly reduced survival of *D. citri* adults during the initial 4 days of evaluation post-treatment as compared to survival observed in both controls (Fig. 6A). Correspondingly, phloem sap collected from CTV-t*Awd*-t*Wnt*-t*PDS*-inoculated plants caused higher cumulative mortality of *D. citri* adults than sap collected from *C. macrophylla* and CTV-wt-inoculated plants; survival did not differ between the two control treatments (Fig. 6B).

## Discussion

HLB is a widespread citrus disease most likely caused by the bacterium, ‘*Candidatus *Liberibacter asiaticus**’. There is a pressing need for more sustainable HLB management methods that extend beyond merely controlling the vectors with insecticide sprays. Our investigation builds toward a biotechnological approach where the cultivated crop is inoculated with a benign and bioengineered virus vector, which expresses dsRNAs that render the inoculated host more appealing, yet lethal, to the attracted phytopathogen vector. In our investigation, citrus plants inoculated with CTV engineered to express the triple construct of t*Awd*-t*Wnt*-t*PDS* were preferred by the vector over wild-type controls in direct choice assays, yet greatly reduced development and survival of psyllids that landed and fed on them. Moreover, a proportion of *D. citri* that did survive on these treated plants exhibited wing malformations caused by the silencing of *DcAwd* and *DcWnt* genes via delivery of dsRNA to the psyllids through the plant phloem. Importantly, delivery of dsRNA via the CTV virus vector eliminates the need for a specialized formulation to apply the dsRNA onto the crop or insect. The virus accumulates and generates significant amounts of dsRNA within the citrus phloem [48], which serves as the sole nutritional source for both immature and mature stages of *D. citri* [12]. Additionally, this well-known ‘mild strain’ of CTV remains stable within the phloem of citrus for extended durations without causing symptoms of disease, expresses foreign genes for up to several years [56], and is relatively fast acting [46]. Therefore, the inoculation of citrus trees with CTV-t*Awd*-t*Wnt*-t*PDS* should be compatible with current HLB management and citrus production practices.

**Figure 7:**
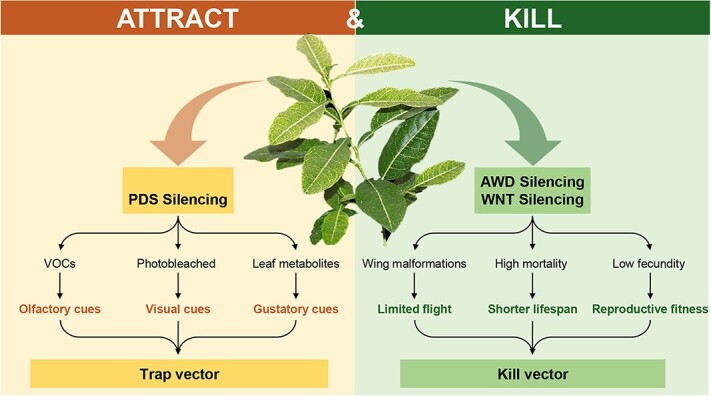
Schematic diagram describing how inoculation of citrus plants with engineered triple gene construct (CTV-t*Awd*-t*Wnt*-t*PDS*) could be deployed to attract and kill *D. citri*. **(I)**  *PDS* silencing results in enhanced olfactory (VOCs), visual (photobleached phenotype), and gustatory (phloem sap chemical composition and leaf metabolites) cues that attract *D. citri*. **(II)** After the insect vector feeds on CTV-t*Awd*-t*Wnt*-t*PDS*-inoculated plants, RNAi-induced silencing of both abnormal wing disc (*Awd*) and wingless (*Wnt*) expression kills vectors or otherwise impairs the ability to transmit pathogen.

Our approach is novel in that three truncated genes were engineered into the CTV vector to create dsRNAs that modified the expression of traits in both the vector’s host plant and the vector itself, yielding a ‘living’ attract-and-kill tool for pest control. The current biotechnological approach of developing attract and kill for *D. citri* by modifying the host plant of the vector offers a potential solution to the problem of density dependence characterizing attract and kill because the entire crop can potentially deliver the ‘kill’ to the target pest. After inoculation of trees with the CTV-t*Awd*-t*Wnt*-t*PDS* triple construct, trees are rendered both hyper-attractive and lethal to the vector yet remain benign to the environment and nontarget organisms because of the high specificity of dsRNA and delivery mechanism (Fig. 7). The current approach also either satisfies or obviates the need for the above-described requirements or limitations characterizing existing attract-and-kill technologies. By silencing *phytoene desaturase* via RNAi in citrus plants inoculated with our CTV triple construct, we express key visual, chemical, and gustatory cues in plants [30] that render these trees hyper-attractive to the vector compared with wild-type hosts (Fig. 7).

RNAi-induced silencing of both abnormal wing disc and wingless pathway signaling genes with the same construct effectively killed or otherwise malformed the vector obviating the need to introduce an additional foreign toxicant into the environment. Previously, Tomaseto *et al.* [57] suggested that a genetically engineered trap crop, capable of interfering with *D. citri* survival, could be planted on the edges of citrus orchards to attract and kill *D. citri*, replacing the need for insecticides. In areas where HLB is only beginning to invade, the trap crop approach could be installed by inoculating only border row trees with the bioengineered CTV. In contrast, in areas where nearly 100% of cultivated trees already express HLB disease, such as Florida [58], it may be necessary to infect all reset trees with CTV-t*Awd*-t*Wnt*-t*PDS* to slow the spread of the disease. These deployment strategies overcome the density-dependent efficacy limitation of attract and kill because large parts of or the entire crop monoculture could function as a killing agent for the vector obviating the need for deployment of many artificial devices designed to mimic the herbivore’s plant host. In either case, the treatment could be deployed relatively quickly in citrus industries impacted by HLB [30]. The currently described biotechnological approach, where existing host trees are inoculated with a benign virus vector to transform them into ‘living’ attract-and-kill devices could both decimate vector populations and extinguish pathogen transmission. Importantly, the technology could reduce insecticide input for *D. citri* management and should be entirely compatible with the large cohort of natural enemies that regulate populations of *D. citri* when insecticide inputs are low [59].

## Conclusion

Beyond the potential application to the HLB crisis, the biotechnological approach described here could revolutionize the idea of using attract and kill for pest control. To date, attract-and-kill technologies have been developed by creating artificial bait stations emitting visual and olfactory cues meant to compete with the authentic crop. Typically, development of such technologies fails either because the natural plant host cannot be outcompeted by the artificial mimic(s) or because the number of devices required per area of crop to impact high population densities of pests sufficiently to achieve crop protection is economically unfeasible [43]. Rather than competing with the cultivated crop, the novel ‘living’ attract-and-kill method introduced here transforms the crop into a hyper-attractive population sink for the unwitting herbivore pest.

## Material and methods

### Insect colonies

Colonies of *D. citri* were continuously reared on pathogen-free *Bergera koenigii* trees within insect-proof mesh cages in a USDA-APHIS/CDC-approved secured growth room at the Citrus Research and Education Center (CREC), University of Florida, Lake Alfred, FL, USA. Psyllids were maintained at 27 ± 1°C, with 65 ± 2% relative humidity (RH) and L16:D8 h photocycle. Throughout this study, newly emerged psyllids (~3 days old) were collected using an oral aspirator (Bioquip, Rancho Dominguez, CA, USA) without discrimination by sex, size, or color.

### Plant materials

*Citrus macrophylla* (commonly known as Alemow) was used as the experimental model plant. The Alemow plants were ~2 ft in height with pencil-thick stems and ~1 year old. All experimental plants were maintained in an approved USDA–APHIS/CDC-secured greenhouse at 24 ± 1°C, 65 ± 2% RH, and L16:D8 h photocycle at the CREC. Plants were watered twice weekly and fertilized once using 20–10–20 NPK fertilizer (Allentown, PA, USA). CTV-wt and CTV-t*PDS* plants were produced previously by Killiny *et al*. [60] via graft inoculation. Pathogen-free *C. macrophylla* was used as a negative control comparison.

### Generation of CTV-t*Awd*-t*Wnt*-t*PDS* plants

The infectious cDNA clone of *citrus tristeza virus* (CTV isolate T36; GenBank accession no. AY170468) in the binary vector pCAMBIA-1380 (Fig. 1A) was used as a base plasmid for engineering all constructs used in this study [56, 61–63]. This plasmid is referred to as wild type (CTV-wt), and it contains full-length cDNA composed of CTV genomic RNA as described by Hajeri *et al*. [48]. PacI (+) and as-StuI (−) were engineered as specific restriction sites to ligate the insert’s under coat protein (CP) subgenomic RNA controller element (CE) between ORF-p23 and the 3′-untranslated regions. Primers used to clone the truncated t*Awd*-t*Wnt*-t*PDS* regions are listed in Table S2. Briefly, a G-block of the truncated fragment consisted of nine nucleotides in total and was composed of *Awd* (263 nucleotides), followed by *Wnt* (313 nucleotides), and then *PDS* (392 nucleotides) and PacI and StuI restriction sites (14 nucleotides) (Fig. S1). This was used as a template for the polymerase chain reaction (PCR). The PCR product was treated with *PacI* (+) and as-*StuI* (−) restriction enzymes, and then inserted into a CTV-wt vector that had been engineered to contain CTV CP CE and specific PacI and StuI sites [48]. The CTV-wt and CTV-t*Awd*-t*Wnt*-t*PDS* constructs were then agro-infiltrated into *Nicotiana benthamiana* for propagation; the virion was subsequently isolated and bark flap inoculated into *C. macrophylla* seedlings as described by Gowda *et al.* [63].

### Validation of small RNA production in CTV-t*Awd*-t*Wnt*-t*PDS* plants

To validate the production of small RNA within CTV-t*Awd*-t*Wnt*-t*PDS* plants, total RNA was extracted from 1 g of leaf tissues according to the method described by Chomczynski and Sacchi [64], then adjusted to 25% ethanol to enrich the small RNA fraction. Subsequently, the RNA samples were filtrated using glass fiber filters to separate and collect the small and large RNAs, as described by Hajeri *et al*. [48]. Northern blot was used to detect the siRNAs according to the procedure detailed in the mirVana™ miRNA Isolation Kit (Life Technologies Corp., Thermo Fisher Scientific, CA, USA) with slight modifications [48]. The cDNA sequence of t*Awd*-t*Wnt*-t*PDS* was cloned into the pGEM^®^-T Easy Vector System (Promega Corporation, Madison, WI, USA), and negative-stranded DIG-labeled riboprobes were produced using the DIG RNA Labeling Kit (Roche Applied Science, Penzberg, Germany) and T7 RNA polymerase. These probes were further hydrolyzed into 50–100 nt-long RNA pieces by treating them with sodium carbonate buffer as described by Dalmay *et al*. [65]. Prehybridization and hybridization were both done at 41°C using 10 ml of ULTRAhyb™ Ultrasensitive Hybridization Buffer (Thermo Fisher Scientific, Portsmouth, NH, USA) per 100 cm^2^ of the membrane. The rest of the Northern protocol was followed as described previously [61], except for a high stringency wash at 41°C. Synthetic 5′-DIG-labeled oligonucleotides of 18 and 21 mer, which ran as 20 and 22 nucleotides, respectively, were used as siRNA size markers in small RNA Northern blot hybridizations.

### Expression of *Awd*, *Wnt*, and *PDS* genes in *D. citri*

*Diaphorina citri* were continuously reared and fed on noninoculated, CTV-wt-inoculated, or CTV-t*Awd*-t*Wnt*-t*PDS*-inoculated *C. macrophylla* plants for 2 weeks. Thereafter, psyllid adults were collected (five insects per plant) and total genomic RNA was extracted using TriZol^®^ reagent (Ambion^®^, Life Technologies, Austin, TX, USA). The quality and quantity of isolated RNA were verified using a NanoDrop 2000 spectrophotometer (Thermo Fisher Scientific, Waltham, MA, USA). Subsequently, a SuperScript first-strand synthesis system (Invitrogen, Carlsbad, CA, USA) with random hexamer primers was used to synthesize cDNA following the manufacturer’s procedure. SYBER Green PCR master mix (Applied Biosystems, Foster City, CA, USA) was used to perform the quantitative PCR (qPCR) using an ABI 7500 Real-Time PCR System (Applied Biosystems, Waltham, MA, USA). Samples were analyzed in triplicate for each biological replicate (five biological replicates per treatment). Primers used to measure the expression of targeted genes (*Awd*, *Wnt*, and *PDS*) are listed in Table S2. The relative expression of tested genes was determined according to the 2^-ΔΔCT^ method [66]. *Actin* was used as the reference gene to normalize collected data; *GFP* was used as an irrelevant gene, whereas *α-tubulin* was used as a nontarget gene.

### *Diaphorina citri* attraction/preference assay

Host preference of *D. citri* was assessed using a no-choice assay with noninoculated, CTV-wt-inoculated, or CTV-t*Awd*-t*Wnt*-t*PDS*-inoculated *C. macrophylla* as described by Johnston *et al.* [67] with slight modifications as described in our previous study [30]. Briefly, two plant treatments (CTV-t*AWD*-t*WNT*-t*PDS* vs. CTV-wt or CTV-t*AWD*-t*WNT*-t*PDS* vs. *C. macrophylla*) were positioned at random at opposite corners of a 60 × 60 × 90-cm insect-proof mesh cage (#1466CV, BioQuip Products, Rancho Dominguez, CA, USA). Subsequently, 100 newly emerged *D. citri* adults were collected into separate 50-ml vials per replicate and released in the center of each cage after removing the vial cap. The cages were maintained under the environmental conditions described for rearing above. After 24 hours, the numbers of *D. citri* settling per plant were counted. The settling behavior of *D. citri* adults is influenced by olfactory and visual cues. Therefore, an identical experiment was also conducted under complete darkness to eliminate the role of visual cues. The materials and procedures were otherwise as described above under light conditions. The entire experiment was repeated six times under light conditions and three times under dark conditions.

### Developmental progression of *D. citri* on CTV-t*Awd*-t*Wnt*-t*PDS* plants

*Diaphorina citri* were sexed and then transferred as male and female (1:1) pairs onto noninoculated *C. macrophylla* plants with new leaf flushes to promote oviposition. Subsequently, 100 newly emerged *D. citri* adults (2–3 days old) were transferred, reared, and fed on noninoculated, CTV-wt-inoculated, or CTV-t*Awd*-t*Wnt*-t*PDS*-inoculated *C. macrophylla* plants within insect-proof mesh cages in a USDA-APHIS/CDC-approved secured growth room for 2 weeks as described above. Afterward, 100 newly emerged F_1_ adults were re-released onto plants to allow feeding, and the number of eggs laid by adults from the second generation was counted for an additional 2 weeks. The entire experiment was repeated twice, with 100 psyllids (50 males and 50 females) per replicate and five replicates per treatment. After 2 weeks, the developmental progression of *D. citri* was determined as follows.

#### Adult mortality

The number of surviving and dead adults was counted to determine the percentage of adult mortality. *D. citri* found on their sides or back and unable to move when prodded with a camel-hair brush were counted as dead.

#### Fecundity

Fecundity was calculated by counting the total number of eggs laid by *D. citri* females per plant per treatment for 25 days using a stereomicroscope.

#### Egg fertility

Egg fertility (defined as the percentage of eggs that successfully hatched following oviposition) was determined as described previously [68]. Briefly, individual detached leaves with eggs were placed into 9-cm petri dishes containing 1.5% agar medium covered with moisturized filter paper to prevent egg desiccation and maintained under the rearing conditions described above. Egg viability was determined by counting and removing nymphs that successfully emerged from eggs every day until no nymphs appeared. This was conducted for both the first and second generations using a 10× magnification under Leica Wild M3Z Stereozoom Microscope (Leica Microsystems, Wetzlar, Germany).

#### Nymph mortality

Nymph mortality (for two successive generations) was recorded every 3 days. Counts were pooled for first and second as well as third through fifth instars. Nymphs found on their sides or back and unable to move when prodded with a camel hairbrush were considered dead. The experiment continued until all psyllids emerged as adults or died.

#### Adult emergence

The number of emerging adults that survived and appeared fully viable on CTV-t*Awd*-t*Wnt*-t*PDS*-inoculated plants or both controls (noninoculated *C. macrophylla* and CTV-wt-inoculated) was recorded for both first and second generations.

### Adult survival over time

Newly emerged psyllids (*n* = 50 per replicate, and five replicates per treatment) were collected from the main colony using an oral aspirator (Bioquip) without discrimination based on sex, and then released on CTV-t*Awd*-t*Wnt*-t*PDS*-inoculated or control (noninoculated *C. macrophylla* and CTV-wt-inoculated) plants. Psyllids were allowed to feed on plant treatments for 2 weeks under the rearing conditions described above. All eggs found during insect counts were removed. Survival was quantified for two successive generations. Briefly, the survival of the released F_0_ adults was recorded every 3 days. Surviving F_1_ adult psyllids (progeny of the first generation) were collected from each treatment plant throughout the experiment. These F_1_ adults were then re-released onto CTV-t*Awd*-t*Wnt*-t*PDS*-inoculated or control plants (100 insects per plant) for a subsequent 2-week exposure interval to evaluate survival daily. Adults *D. citri* found on their sides or back and unable to move when prodded with a camel-hair brush were considered dead.

### Wing malformation

*D. citri* were continuously reared and fed on noninoculated, CTV-wt-inoculated, or CTV-t*Awd*-t*Wnt*-t*PDS*-inoculated *C. macrophylla* plants for 2 weeks as described above, and then fourth to fifth instar nymphs were collected (10 nymphs per plant, 5 plants per treatment) and observed every 24 h until adult eclosion as described previously [47]. Newly emerged adults were collected, and the morphology of forewings and hindwings was inspected for possible malformation under Leica Wild M3Z Stereozoom Microscope (Leica Microsystems) and photographed using a Canon Power Shot S3IS digital camera.

### Survival of *D. citri* on phloem sap

Phloem sap from noninoculated, CTV-wt-inoculated, or CTV-t*Awd*-t*Wnt*-t*PDS*-inoculated *C. macrophylla* plants was collected via centrifugation as described previously [69]. Subsequently, a 0.2-μl droplet of collected phloem sap was topically applied to the ventral side of the thorax of fifth instar *D. citri* nymphs using a 10-μl Hamilton syringe as described previously [70]. Treated nymphs were given 60 seconds to acquire the droplet by feeding. Mortality of *D. citri* adults was quantified as described above for 6 days after treatment. Five biological replicates (20 insects per replicate) were tested per treatment, and the experiment was repeated three times.

### Statistical analysis

All experiments, unless otherwise stated, were conducted using a completely randomized design with five biological replicates per treatment. Data were subjected to analysis of variance followed by the Tukey honestly significant difference (HSD) tests for *post hoc* pairwise comparisons (*P* ≤ 0.05). Survival analysis was conducted using the Kaplan–Meier method [71] to describe the probability of psyllid survival on various plant treatments. *P* values and *χ*^2^ of log-rank and Wilcoxon tests were also calculated and presented within the survival plots for statistical comparisons between treatments. Finally, survival data were also modeled with the Weibull Fit with least squares method.

### Accession numbers

- CTV isolate T36: GenBank accession no. AY170468.
- *AWD*: *D. citri* nucleoside diphosphate kinase-like (LOC103518886), mRNA. Sequence ID: XM_008483968.3.
- *Wnt*: *D. citri* protein wingless (LOC103521460), mRNA. Sequence ID: XM_008486571.2.
- *PDS*: Citrus clementina phytoene dehydrogenase, chloroplastic/chromoplastic (LOC18031616), mRNA. Sequence ID: XM_006420997.2.

## Supplementary Material

Web_Material_uhae311

## Data Availability

All relevant data that support the findings of this study can be found within the article.
