# Supplementary material for: Virus-induced gene silencing simultaneously exploits ‘attract and kill’ traits in plants and insects to manage huanglongbing
Source: Hortic Res. 2024 Nov 6;12(2):uhae311. doi: 10.1093/hr/uhae311 (PMC11817989; doi:10.1093/hr/uhae311)
Supplement: Web_Material_uhae311 [file web_material_uhae311.zip › Attrack and Kill Additional File for Review.docx]

**Table S1. Weibull Parameter Estimates**

| **Treatment** | ***α*** | | | ***β*** | | |
| --- | --- | --- | --- | --- | --- | --- |
|  | **Estimate** | **Lower 95%** | **Upper 95%** | **Estimate** | **Lower 95%** | **Upper 95%** |
| *C. macrophylla* | 37.43 | 34.72 | 40.32 | 2.23 | 1.94 | 2.55 |
| CTV-wt | 36.80 | 34.13 | 39.63 | 2.24 | 1.94 | 2.56 |
| CTV-tAwd-tWnt-tPDS | 13.74 | 12.66 | 14.89 | 2.08 | 1.82 | 2.38 |

same as Extreme-Value with α=exp(λ), β=1/δ

**Table S2. Primers used in this study**

| **gBlock/Gene** | **Purpose** | **Primer** | **Sequence** | **Amplicon size** |
| --- | --- | --- | --- | --- |
| *Awd-Wnt-PDS* | Amplification before insertion into the CTV infectious clone | *Awd-*PacI-F | CGAGT***TAATTAA***CGCTTTGAAGACAAAGGCTTCAAATTGGTG | 991 nt |
|  |  | *PDS-*StuI-R | GACA***AGGCCT***GTCTCATACCAGTTCCCGTCCCCATC |  |
| *Dc-Awd* | Gene expression | *awd*-RT-F | AGAGGACTTGTGGGAAACATC | 200 nt |
|  |  | *awd*-RT-R | TGACAAGACCAGGGAAGAAAG |  |
| *Dc-Wnt* | Gene expression | *Wnt*-RT-F | AACACACGCTTAGCACGAAT | 202 nt |
|  |  | *Wnt*-RT-R | GATCCAGACATGCCGTGACA |  |
| *Cs-PDS* | Gene expression | *PDS*-F | GTCAAAACGCCAAGGTCTGT | 197 nt |
|  |  | *PDS*-R | GCAGCAAGCAGCACATAGTC |  |


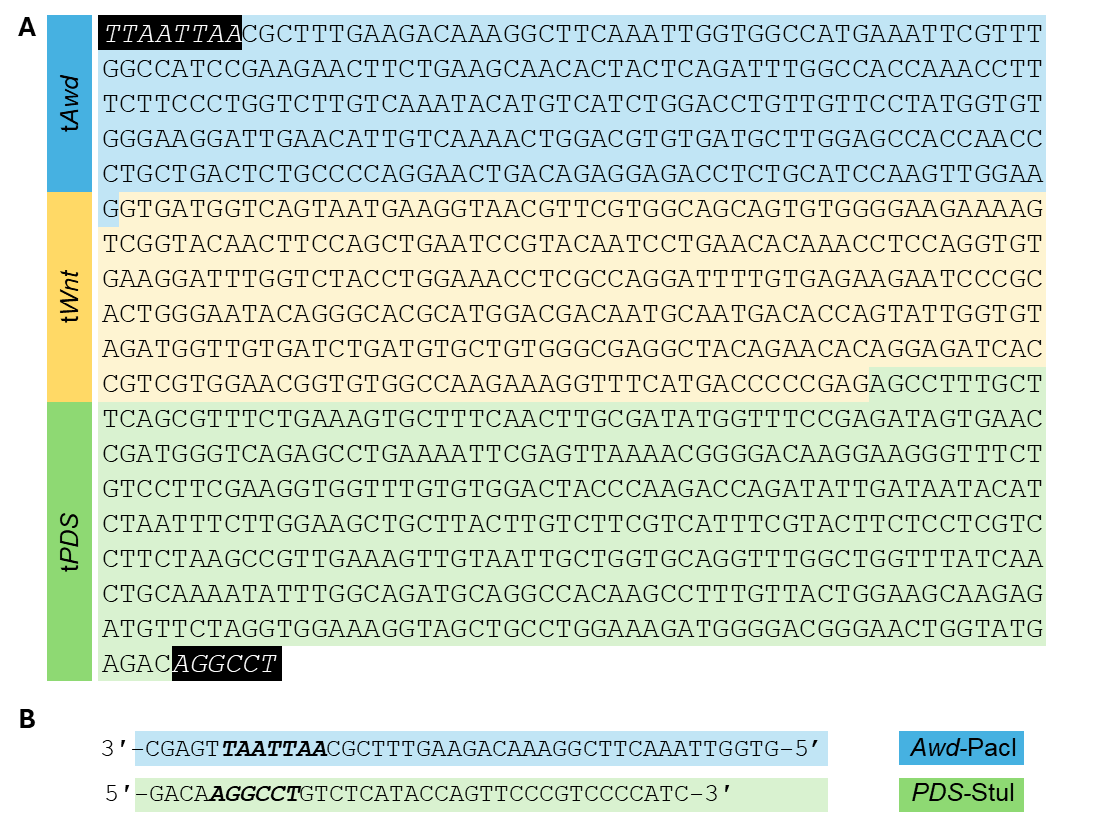


**Figure S1. Sequence of the triple gene construct, CTV-t*Awd*-t*Wnt*-t*PDS* (A) and primers used for amplification (B).**
